# Editorial: Current progress in mesenchymal stem/stromal cell research, volume II

**DOI:** 10.3389/fcell.2023.1177662

**Published:** 2023-03-13

**Authors:** Lindolfo da Silva Meirelles, Darius Widera, Marcela F. Bolontrade

**Affiliations:** ^1^ Graduate Program in Cellular and Molecular Biology Applied to Health—PPGBioSaúde and School of Medicine, Lutheran University of Brazil, Canoas, Brazil; ^2^ Stem Cell Biology and Regenerative Medicine Group, School of Pharmacy, University of Reading, Whiteknights, Reading, United Kingdom; ^3^ Institute for Translational Medicine and Biomedical Engineering (IMTIB) CONICET - Italian Hospital of Buenos Aires, Buenos Aires, Argentina

**Keywords:** mesenchymal stromal cells, mesenchymal stem cells, MSCs, basic research, MSC therapies, MSC-derived extracellular vesicles (EVs), MSC immunomodulation

Currently, mesenchymal stromal cells (MSCs), which are often still referred to as mesenchymal stem cells, are among the most used cells in clinical trials. When the Editorial for the first edition of this Research Topic was written (mid-January 2021), a search at www.clinicaltrials.gov using the string “MSCs OR mesenchymal stem cells OR mesenchymal stromal cells” returned 10,407 registered clinical trials, 3,467 of which were active. Now, as the Editorial for the second volume of this Research Topic is released (early March 2023), the same search returns 13,121 studies, 4,170 of which are active. To give an idea of what those numbers represent, when the same database is queried using the string “HSCs OR hematopoietic stem cells OR hemopoietic stem cells”, it returns 6,096 studies, 1,701 of them active. In spite of the impressive number of clinical trials using MSCs, the use of these cells in the clinic has not become established as a routine yet, which calls for further investigation of their biological properties.

The aim of the second volume of this Research Topic was to provide a venue for high quality research articles and reviews that update the current knowledge on the biology of MSCs, including mechanisms that can affect their role in cell therapy and tissue engineering. Manuscripts submitted to this Research Topic were expected to contain information that can be used to better understand how MSCs behave, and how they exert their therapeutic properties, whether such information comes from *in vitro* or *in vivo* studies. Consequently, suggested subjects for submitted manuscripts included a) novel features of MSCs from different tissues; b) molecular aspects of MSC differentiation; c) Processes for the enhancement of MSC differentiation and expansion in culture; d) identification of MSCs, or cells that can give rise to them, *in situ*; e) acellular MSC-derived products such as extracellular vesicles; f) immunomodulatory properties of MSCs; and g) molecular interactions between MSCs and other cell types *in vitro* or *in vivo*. This Research Topic received 12 manuscripts. Seven of these manuscripts were accepted; four of these were original research reports, and three were reviews. Short descriptions of the papers that make up this Research Topic are provided below.

Ceccarelli et al. examined the effects of 5-azacytidine, a demethylating agent, on proliferation, clonogenicity, migration and adipogenic potential of human adipose-derived MSCs (ASCs), and observed that this agent impairs cell proliferation and stimulates adipogenic differentiation; further experiments suggested that treatment of ASCs with 5-azacytidine impaired phosphorylation of Akt and ERK and that this could be responsible for reduced cell proliferation and migration. In addition, inhibition of the Wnt pathway could be involved in the enhanced adipogenic differentiation observed. Behm et al. studied the effect of 25-hydroxyvitamin D_3_, the major metabolite of vitamin D3 in the blood, on human periodontal ligament-derived MSCs; they found that this molecule diminishes the ability of MSCs treated with interleukin-1 beta to suppress the proliferation of CD4^+^ T cells, but does not have the same effect in the presence of tumor necrosis factor. Findings by Gaggi et al. suggested that human amniotic fluid-derived MSCs could generate motoneurons-like cells. Briefly, the authors induced differentiation by exposing the cells to a sequence of SMAD, ALK2, GSK3, and gamma secretase inhibitors in combination with neurotrophic factors, ascorbic acid and retinoic acid and assessed the resulting changes in gene expression. Moreover, they assessed the formation of neuromuscular junctions and the ability of the differentiated cells to induce skeletal muscle contraction. Overall, the results suggest that amniotic fluid-derived MSCs could differentiate into functional motoneuron-like cells *in vitro*. If validated *in vivo*, these finding could have implications for future translational work focus of conditions affecting motoneurons, such as amyotrophic lateral sclerosis (ALS). Umrath et al. studied the effects of corticosteroids on jaw periosteum-derived MSCs. More specifically, they focused on the impact of dexamethasone on the osteogenesis, extracellular matrix (ECM), and secretion of pro-osteogenic factors by MSCs. They demonstrated that osteogenic differentiation of MSCs isolated from the jaw periosteum does not depend on the exposure to dexamethasone if the cells are cultivated in a medium supplemented by human platelet lysate. In addition, they confirmed earlier findings that suggest that dexamethasone affects not only global gene expression but also the ECM, and the profile of the secretome. As dexamethasone is known to cause severe side effects, new dexamethasone-free protocols for osteogenic differentiation of MSCs are important for developing MSC-based therapies for bone fractures and conditions such as osteoporosis.

Chetty et al. reviewed the compartments of umbilical cord tissue from which MSCs can be obtained, and discussed the properties of these MSC populations. Koga et al. reviewed roles of extracellular vesicles produced by MSCs on tissue repair and regeneration, and how therapies based on MSC-derived exosomes could be advantageous to treat various conditions. Marson et al. provided insights into the *in vivo* nature of MSCs, reviewing existing evidence on the behavior of pericytes as MSCs. A perivascular location would be conducive to rapid action after injury, favoring wound healing through the action of pro-regenerative macrophages. This opens a venue to explore the use of MSCs and their extracellular vesicles, or activated pericytes at sites of injury, for regenerative medicine strategies through non-classical mechanisms, by promoting an M2-like profile in tissue–resident macrophages. Further, the authors discuss the advantages of using MSC-derived acellular products such as exosomes for the induction of an immunomodulatory and wound-healing profile.

